# Attention Restoration Space on a University Campus: Exploring Restorative Campus Design Based on Environmental Preferences of Students

**DOI:** 10.3390/ijerph16142629

**Published:** 2019-07-23

**Authors:** Ming Lu, Jingwan Fu

**Affiliations:** Key Laboratory of Cold Region Urban and Rural Human Settlement Environment Science and Technology, School of Architecture, Ministry of Industry and Information Technology, Harbin Institute of Technology, Harbin 150001, China

**Keywords:** campus design, restorative environment, environmental preference, perceptual factors, place-mapping, landscape types

## Abstract

Students studying for a long time frequently suffer from attentional fatigue; however, campuses lack specific spaces in which to restore attention. This study aimed to explore the significant perceptual factors related to student selection of landscape types that they perceive as most relaxing on a university campus. To understand the design factors of an attention restoration space, this study examined the preference of students regarding restorative environments on university campuses at six universities in northeastern China using a questionnaire survey (n = 360). Place-mapping revealed the spatial characteristics of the preferences of students for relaxing in the available space. The primary perceptual factors were obtained using correlation analysis and keyword frequency. A relationship model of landscape types and perceptual factors was established using categorical regression (CATREG). Results showed that waterfront spaces have the optimal perceived attention restoration effect, followed by vegetation spaces, courtyard spaces and square spaces. Visibility, accessibility, comfort, recognition and sense of belonging are significant perceptual factors that should be first considered. Moreover, the optimal selection of design factors depends on the interaction of landscape types and perceptual factors. The design implications may assist designers to gain a new perspective on student requirements for a healthy environment.

## 1. Introduction

University students spend most of their time studying on campus, which requires effort and may cause attention fatigue. Reports of universities and colleges worldwide indicate the outbreak of mental health problems among college students. According to data statistics from the United States in the autumn of 2018, approximately 12.5% of 26,181 college students at 40 universities felt tired, stressed out or sleepy during the previous 7 days [1] and 29.5% of them had experienced overwhelming anxiety over the previous two weeks [2]. In his book *The Stressed Years of Their Lives*, Dr. Anthony Rostain stated that current college life may be more stressful than ever before [3]. Therefore, to provide students with an effective approach to appropriately alleviate mental fatigue is a pressing concern.

To restore attention through the exposure to the environment is one effective approach [4]. Frequent visits to green spaces by university students can improve their overall mood and reduce perceived stress [5]. Therefore, a university campus must not only provide an environment for study but also one that promotes physical and mental health. However, the landscape types of universities are generally divided into core landscape areas, memorial spaces, square spaces and courtyard spaces [6], which are designed to reflect the campus culture and project the image of the university [7]. Therefore, designers must be attentive to the health requirements of the primary users of the campus and must incorporate a restorative environmental design into the campus design.

Recent studies have examined restorative environments for students primarily from two aspects, namely the naturalness of the physical environment and student activities, through comparing the vegetation space and bare ground or walls [8,9], preferred restorative activities [10] and active or passive participation [5]. Although the attention restoration theory (ART) proposes four factors by which to evaluate a restorative environment [11], relevant studies have focused more on the psychological aspect and lack of the transformation in characteristics of the physical environmental aspect. In general, the environmental factors for attention restoration have been less studied and a design guide is not available to instruct how to create a space on a university campus.

The purpose of this study was to examine the primary perceptual factors associated with selecting the most relaxing landscape type on a university campus. To meet this aim, we addressed the following research questions: (1) What type of landscape has more perceived attention recovery effect? (2) Which perceptual factors have a significant effect on the choice of campus space? (3) Which factors should be considered before designing a restorative campus space? To answer the aforementioned questions, the concept of an “attention restoration space,” which can help people to relax and can relieve stress, restore attention and improve work and study efficiency, was proposed. To relate perceptual factors with environmental design elements, a questionnaire was designed to obtain information on landscape type preferences and spatial perceptual factors preferred by students. To explore the influence of landscape types and perceptual factors on the design of the attention restoration space, regression modeling with categorical regression (CATREG) analysis was employed. Therefore, each factor was ranked and suggestions for the optimal design were elicited. The results can enrich the ART, provide a practical reference for designers and provide an approach to improve the construction of university campuses.

## 2. Materials and Methods

### 2.1. University Samples

Campus samples were six universities in northeastern China that offer different majors, were built at various times and have various landscape types (Table 1). To avoid the bias of one particular landscape preference predominating at one campus, the sample campuses were selected because they were representative of various university types and landscape designs. The study considered the six campuses as a complete sample through which to obtain common environmental characteristics, thus possibly providing a more universal result. The landscape types on campuses typically share common features, although design varies among campuses in accordance with their educational concepts. However, not all universities can afford waterfront spaces because of the limitations of natural resources, financial resources and climate variations. To provide the optimal landscape selection, sample 4 was used only as a contrast sample for comparing the environmental preferences between campuses with or without waterfront spaces; however, this approach did not affect the common features of the complete sample.

### 2.2. Landscape Types of University Campus

Nowadays, campus environments are park-like spaces, which include diverse landscape types that can be categorized as natural and artificial in general. Studies have reported that some specific environmental features contribute to the attractiveness of landscapes, such as open meadows surrounded by woods [12], natural water areas [13] and deciduous forests [14]. Therefore, the campus environments were subdivided to examine the effect of different landscape types on the spatial selection of attention restoration spaces [15]. The conventional landscape types of a university campus were classified based on the dominant landscape elements according to area metrics, such as plants, water bodies, pavement and landscape structures [16]. Area metrics can be used to analyze different landscape types based on the percentage of landscape area [17], which is one of the indicators of the landscape structure index [18]. First, the open space on campuses was selected from several blocks based on the OpenStreetMap. Second, different landscape elements were calculated using the area percentage. Finally, landscape types on the campuses were validated through field investigation and defined using their dominant area percentage.

**Figure 1:**
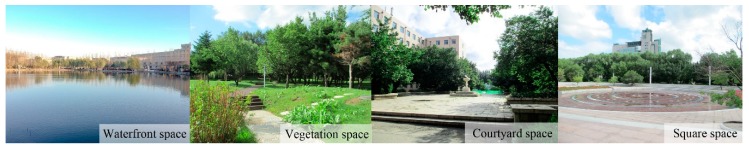
Examples of landscape types of university campus (from sample 3).

In this study the four types of landscape were subdivided as waterfront spaces, vegetation spaces, courtyard spaces and square spaces (Figure 1). Waterfront spaces are dominated by water bodies, such as fountains, pools, rivers and lakes. Vegetation spaces are occupied by plants, such as lawn space (e.g., grass, flowers and bushes) and forest spaces are dominated by woods (e.g., combination of trees, shrubs and grass). Square spaces primarily comprise pavement with a large open view. Furthermore, square spaces are a conventional landscape type at Chinese university campuses, which are designed as the representative image of the university and squares tend to be an indispensable venue for all types of ceremonies and large-scale activities. Courtyard spaces emphasize the space enclosed by buildings and have plants and landscape structures, such as pavilions and galleries. This study did not, however, consider playfields because attention is primarily restored through visual perception.

### 2.3. Perceptual Factors

Perceptual factors reflect student perceptions of the external environment related to attention recovery. The selection of perceptual factors was based on the ART and preference matrix, which provided a basis for investigating the relationship between the restorative effect and environmental preference. ART proposes four components for the restorative environment, namely “being away”, “fascination”, “compatibility”, and “extent” [11]. Being away indicates psychological distance from daily routines that leads to directed attention. Fascination refers to many landscape elements, such as water, vegetation and butterflies, which can effortlessly draw attention and help people relax. Compatibility means environmental settings for activities, such as meditation, hiking and bird watching. Extent is the sense of a small space offering a boundless sense of environment. Furthermore, four components were included in the matrix, namely coherence, complexity, legibility and mystery [19]. Coherence refers to the environmentally salient elements that can be easily distinguished. Complexity means the diversity of the environment and the availability of numerous things to explore. Legibility provides people clear landmarks to find indications easily. Mystery indicates cues to explore more into the setting.

Table 2 shows 10 perceptual factors that were summed up according to ART [19,20], which was adjusted according to university campus space features [21] and a review of relevant literature on the restorative environment [22,23]. Because the campus environment is a combination of natural and artificial landscapes, the restorative factors from the ART and preference matrix were modified to improve their applications in this research. Legibility was translated as recognition to reflect on the layout. Coherence was translated as familiarity of the design style. Extent and fascination were expressed as being related to pleasure that makes students feel happy with not only the landscape design but also interesting activities in a space. Compatibility was conveyed as a sense of belonging; for instance, by providing shelters and spaces to adapt to diverse activities. Being away was conveyed as a calm environment, which could provide a peaceful place. Complexity and mystery were understood to be reflected in landscape design that has attractive features. Furthermore, four factors were supplemented to evaluate the restorative campus. Accessibility was explained as the ease of reaching restorative spaces [24]. Visibility was defined as the ease of seeing restorative views [25]. Comfort was represented as the campus infrastructure and suitable landscape maintenance [26]. Creativity was presented as a space aimed to stimulate creative thinking; this concept was based on previous studies that have reported a positive relationship between the academic performance and campus green space [27,28].

### 2.4. Questionnaire Design

A self-reporting questionnaire is a commonly used method in environmental psychology [29]. The questionnaire survey was conducted with field observation. The questionnaire was designed on the basis of environmental preferences to select the primary design factors for an attention restoration space. Observation was used for the preselection of campus landscape types and as a support for the questionnaire design. The landscape types of the campus and behavior characteristics of the students were recorded through field observation. Self-reporting questionnaires were randomly distributed among students.

The title of the questionnaire is “university outdoor restorative environment survey for relaxing your mental fatigue.” The questionnaire comprised four parts. The concept of an attention restoration space was first explained to the students. The campus map and pictures of different landscape types, specifically at that campus, were attached. The demographic questions were asked at the end of the questionnaire and these solicited information regarding gender, age, major and education. (1) To understand the characteristics of different landscape types, marking a spot on a map was selected as an approach to visualize and find a particular space [30]. The participants were required to circle the place where they generally relax on the campus map, excluding the playfield; (2) The students were asked “how satisfied are you with the selected space as an attention restoration space?” The satisfaction level and overall performance of 10 perceptual factors were evaluated using a 1–5 point scale (1 point = not satisfied at all, 5 points = very satisfied) according to the selected restorative environment; (3) To obtain the usage patterns, which could reflect how student engage with a space based on their needs, the participants were asked to rank the landscape types and answer each question according to their environmental preference based on their real school life, such as visiting frequency, visiting time, stay duration, activities and whether they tend to have company during the visits; (4) To acquire more information of how to improve the campus outdoor environment, an open-ended question within the questionnaire was included. In this section, each participant who finished the above questions was asked to describe their ideal attention restoration space on campus: “If you want to find a place to relax your brain, what do you think must be improved about your campus environment? Brief descriptions are welcome.” The responses of students were written down to ensure that the participants completed the questionnaire.

### 2.5. Participants

The subjects are students who are living on campus. Convenient sampling was conducted to approach the subjects easily and was not affected the validity [31,32]. The survey was conducted at the main library on each sample campus as it is not only the core area of the campus but also the most frequently visited study space. The distribution location was an indoor or outdoor free talking zone equipped with tables and chairs in the public area, which gave us the opportunity to reach to a variety of students. We randomly approached students who came into or came out of the library during the workdays from 8 a.m. to 9 p.m. during the spring semester and asked if they were willing to participate in a questionnaire and brief interview about the relaxing outdoor environment on campus. Students who agreed to participate were brought to the table where the questionnaire has been prepared, a researcher would guide the participant and interviewed him or her at the last open-ended question.

In this study, 360 valid questionnaires were obtained [33,34]. The participants had different genders, ages, majors and educations, which provided an improved representation of the characteristics of university campus users and provided high heterogeneity. Participants had an age range of 17–36 years (mean age = 21.17, SD = 2.35) from the six universities. They were diverse in liberal arts (37.5%), science (6.1%) and engineering (56.4%), covered 78.6% undergraduate students, 19.7% graduate students and 1.7% doctoral students. Although they were from different universities, the students were considered to be one sample because their campuses shared the same landscape types. Furthermore, students from different campuses could represent the common preference for similar landscapes, which could be evaluated at university campuses other than the samples.

### 2.6. Statistical Analyses

A data-driven approach based on descriptive statistics, correlation analysis and regression analysis was used in this study. All analyses were performed using SPSS 19.0. The Cronbach’s alpha of 10 perceptual factors was 0.859 (>0.7), which was obtained through a reliability analysis and indicated that the results could be statistically analyzed [35]. The environmental preferences were collected through descriptive statistics of place-mapping and self-reported questions. The primary perceptual factors were selected through correlation analysis and keyword frequency. The locations where they generally relax and study were marked on the campus map by the participants. Their answers to the structured questions were analyzed according to the environmental preferences. Correlation analysis was used to preliminarily identify the significant perceptual factors related to spatial usage preference. Keyword frequency identified words with a high appearance rate in the description of participants obtained through the open-ended question, with the ideal environment of the attention restoration space as a supplement to the selection of the primary perceptual factors.

To understand the relationship between perceptual factors and landscape types, CATREG was employed to model the influencing factors of the landscape types, which students perceived to be the most restorative. For modeling the relationship between perceptual factors and landscape types, we applied CATREG using the score of the primary perceptual factors as independent variables (X) and the overall score of recovery degree of selected landscape types as dependent variables (Y). The CATREG model was processed in the following expression [36]:

(1) $$Y=\sum_{i}^{I}\beta \mathrm{iXi}+\varepsilon$$

where *I* is the number of independent variables, Y is the recovery degree of selected landscape types, X_i_ is the primary perceptual factors after selection, β_i_ is the regression coefficient and ε is an error vector. This method could be used to obtain the optimal linear regression equation by assigning different categories of the given data with various types of variables and measure nonlinear relationships [37,38]. In this study, the effect of ordinal categorical variables could be more accurately analyzed and the test results were more reasonable and credible than those of the standard analysis performed using CATREG with optimal scaling. We used CATREG Version 3.0.0., developed by Data Theory Scaling System Group (DTSS), Faculty of Social and Behavioral Sciences, Leiden University, the Netherlands in SPSS 19.0 [39].

## 3. Results

### 3.1. Environmental Preference of Attention Restoration Spaces

#### 3.1.1. Landscape Type Preference

**Figure 2:**
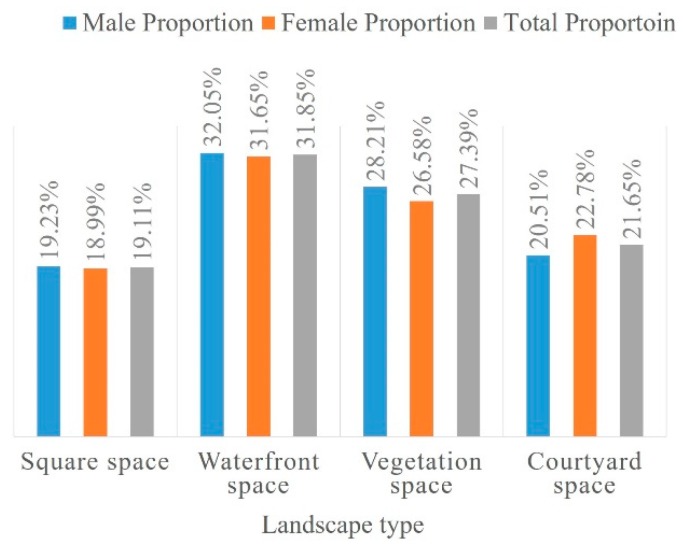
Landscape type preference.

**Figure 3:**
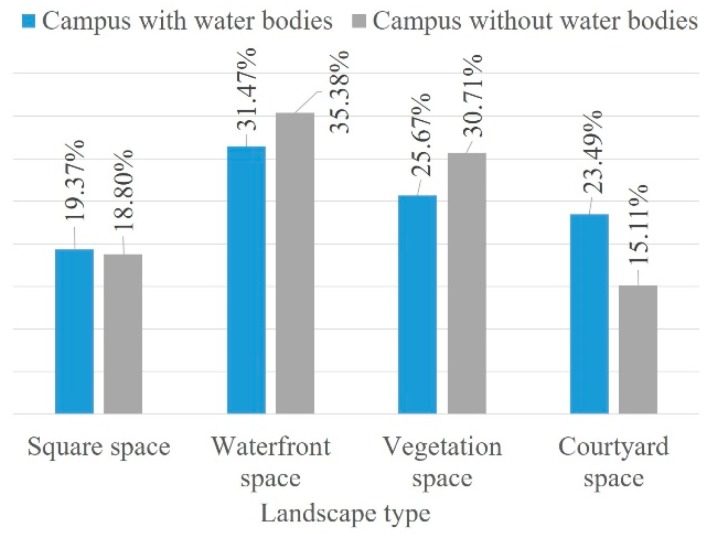
Comparison of waterfront space preference.

The ranking of the landscape types indicated the type of spaces on campus preferred by the students for relaxing. Figure 2 shows no significant difference in landscape type preference between genders. Waterfront spaces were perceived as the most restorative spaces among users (31.82%), followed by vegetation spaces (27.59%), courtyard spaces (21.58%) and squares (19.01%). Furthermore, natural elements (the overall percentage of waterfront and vegetation space was 59.41%) were considered more restorative than artificial elements (the overall percentage of courtyard spaces and squares was 40.59%). In contrast to campuses with water bodies, waterfront spaces were still the first preference among the four types even at campuses without water bodies (Figure 3).

**Figure 4:**
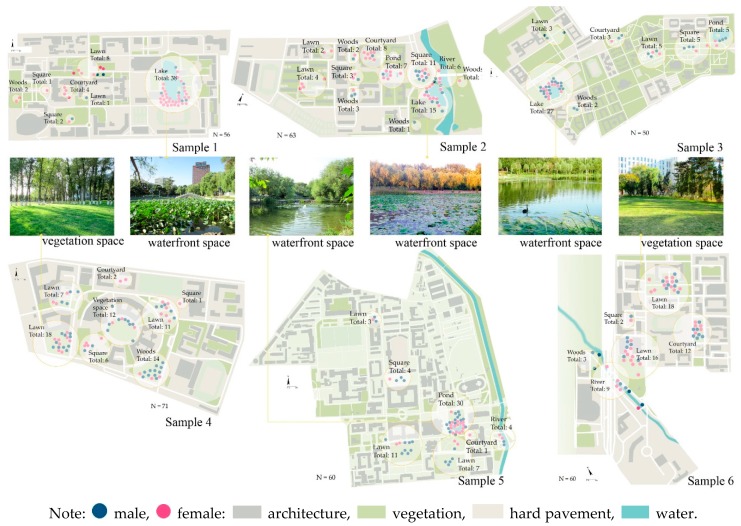
Preference of attention restoration space on university campus and examples of best perceived restorative spaces based on place-mapping.

By marking the location of the attention restoration space on the campus map (place-mapping), the environmental preferences for different spatial characteristics at the real campuses were reflected. Figure 4 shows pictures of the favorite spots of students selected through place-mapping. At the sampled campuses, students preferred to relax in a landscape with large water bodies or lawns and tall trees. In particular, a large water body with a broad view (samples 1, 2, 3 and 5) was considered more pleasant and relaxing compared with a narrow river view (sample 6). The vegetation space was primarily affected by the spatial scale and openness in comparison with other similar spaces, which indicated that large areas of grasslands were more preferred (samples 4 and 6). The courtyard with much greenery and with seats in the shade had the highest utilization rate from the selected spots of all sample campuses, as determined through observation. Furthermore, such areas provided high-quality greenery, recreation facilities and privacy. However, few people visited the spaces with more hard landscapes or those without vision blocks, seats, patio and pavilions provided. The same observations were made for square spaces of all samples; squares with green shades and seats were more preferred than those with only sculptures or those that lacked shelters.

#### 3.1.2. Spatial Usage Preference

To understand the daily requirements of students for attention restoration spaces, spatial usage preferences were examined for the interaction level, being alone or with friends, visiting time preferences, visit frequency and duration preferences to examine the spatial preference.

**Figure 5:**
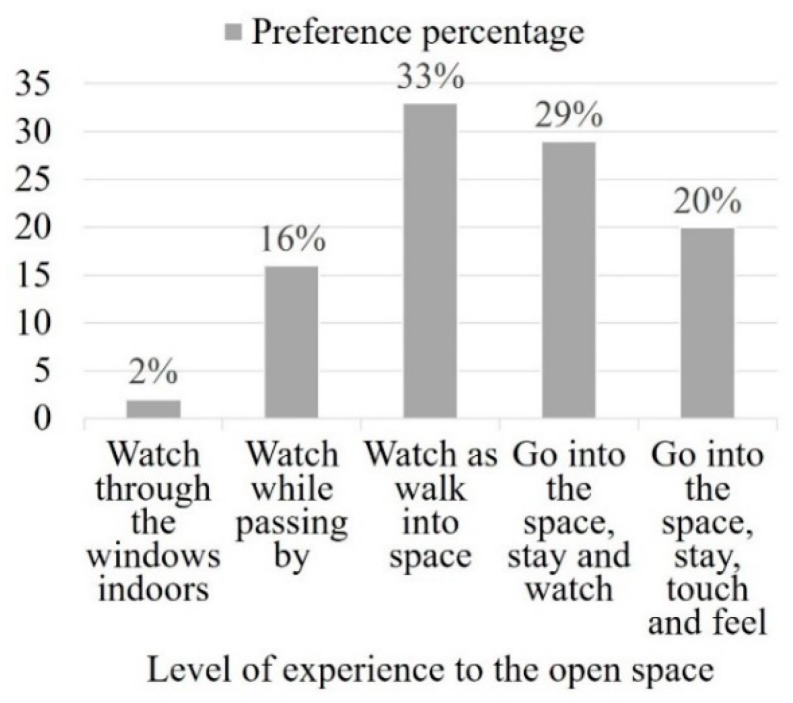
Interaction level of visit preference.

Most of participants (82%) prefer to rest and enjoy the views in the space as an optimal approach to restore themselves; their activities tend to entail watching while walking into the space (33%), going into the space, staying and watching (29%) and going into the space and staying, touching and feeling (20%) (Figure 5). Approximately half of the participants (49%) enjoy staying and interacting with the surrounding environment, doing things such as touching plants and playing with water. Moreover, the participants reported that a suitable infrastructure and natural materials could increase the attractiveness of spaces.

**Figure 6:**
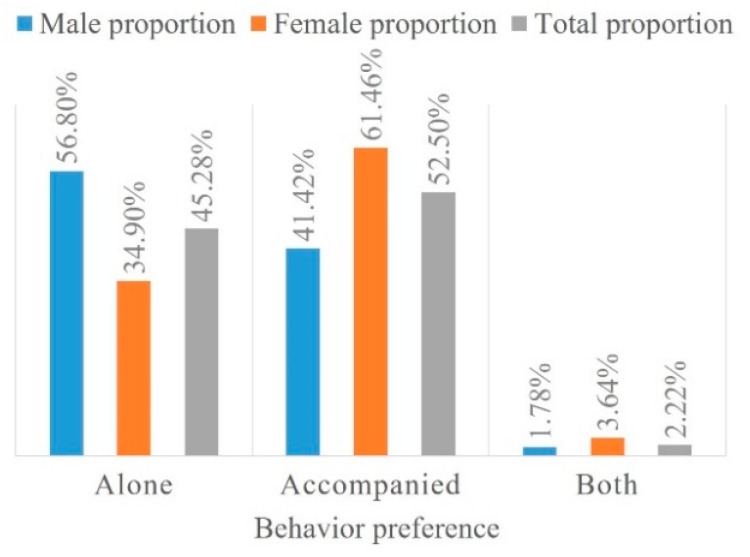
Being alone, accompanied or both.

When visiting restorative spaces, 45.3% of participants preferred to be alone, 52.5% preferred to be accompanied and the remaining participants preferred both (Figure 6). Male students constituted 56.8% of the participants who preferred to be alone, which indicates a slightly higher preference for privacy than that of females. However, female students were more likely to walk with friends than male students. This result suggests that private spaces are equally vital to open spaces on campuses.

**Figure 7:**
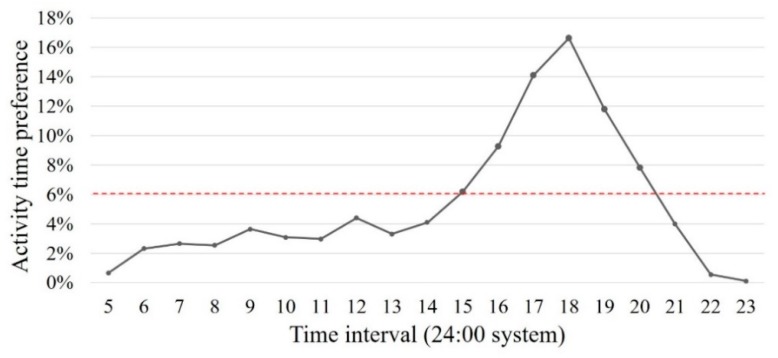
Time preference of visit.

The peak visiting hour shows most students (66%) would like to take a rest around noon and in the afternoon, from 3 p.m. to 8 p.m., especially after the dinner time around 6 p.m. (Figure 7). The rest of the participants visited the restorative space from 5 a.m. to 2 p.m. and after 8 a.m. This result suggests that students usually visit the restorative space after class according to the class hours.

**Figure 8:**
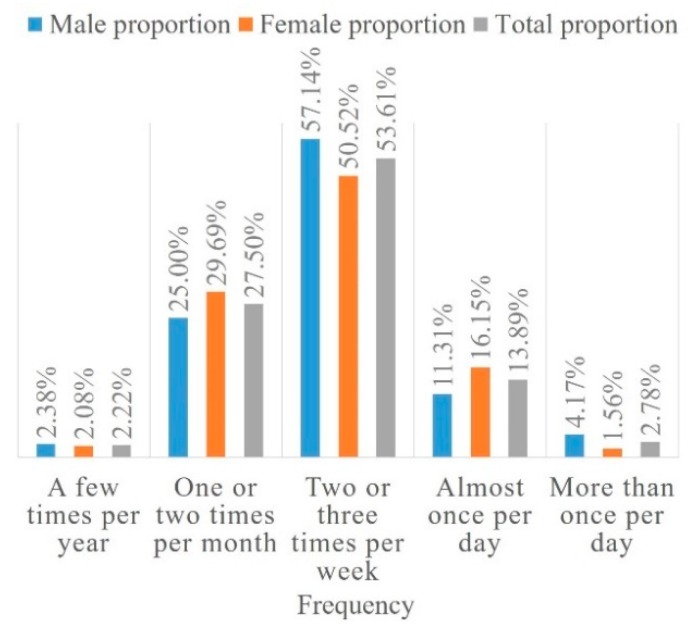
Frequency preference.

Figure 8 shows that approximately 17% of the participants went to restorative spaces at least once a day. More than half of participants went to the restorative spaces two or three times a week. Approximately 30% of participants seldom went to a restorative space, with a frequency of once or twice a month and a few times a year.

**Figure 9:**
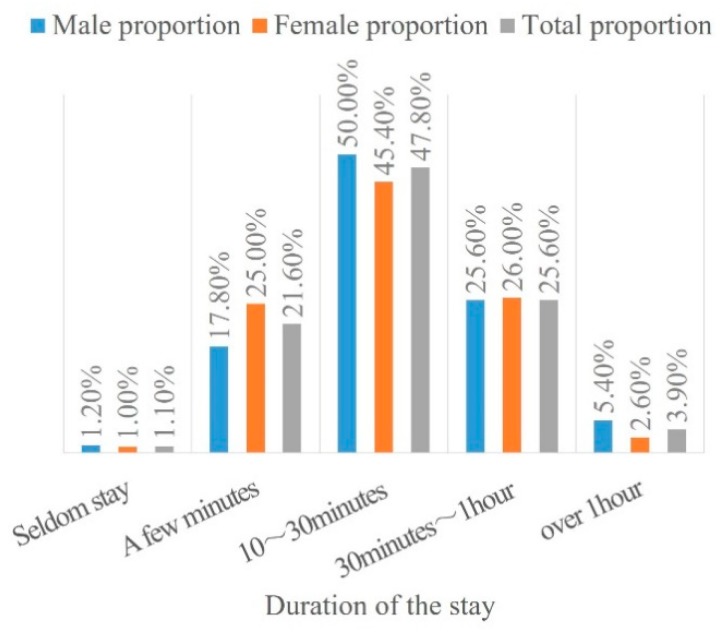
Staying time preference.

Figure 9 shows that more than 77% of the participants were willing to stay for more than 10 minutes in their selected space, whereas approximately 22% of the participants preferred to enjoy the sights when passing by for only a few minutes.

Overall, the statistical results showed that the majority of the participants preferred to relax in outdoor spaces (more than 70%) and for visit durations longer than 10 minutes, thus indicating that open spaces on campuses have excellent potential for attention recovery. Majors and education levels of participants were not significant for environmental preference.

### 3.2. Perceptual Factors of Attention Restoration Space

The primary perceptual factors of attention restoration spaces were validated and selected through correlation analysis and keyword frequency statistics. Correlation reflects the primary perceptual factors of students’ priority when choosing a space of their campus. Keyword frequency was supplemental to the perceptual factors based on the ideal features of student requirements.

#### 3.2.1. Primary Perceptual Factors

The frequency and average time spent at the perceived restorative spaces reflect the popularity of the space. The students visited their restorative spaces more frequently and stayed for longer durations when the space had suitable perceptual factors. Therefore, correlation analysis was used with these two factors to examine the significant perceptual factors. Analysis using the Spearman rank correlation coefficient [40,41] showed that the frequency of using these spaces was significantly correlated with accessibility, visibility, recognition and sense of belonging, whereas the average time spent in such spaces was significantly correlated with comfort (Table 3). Among the significant factors, recognition was the most relevant factor, followed by sense of belonging and visibility, with *p* < 0.01. Furthermore, accessibility and comfort were significant, with *p* < 0.05.

The landscape type and the method of utilization may affect the correlation results and may reduce the significance of certain perceptual factors. For example, waterfront spaces may not be affected by the distance obtained by mapping the campus, as the water bodies are often located far away from the primary study buildings. Therefore, a further analysis using the keyword frequency from the interview was used to help provide an improved perspective.

#### 3.2.2. Ideal Perceptual Factors

**Figure 10:**
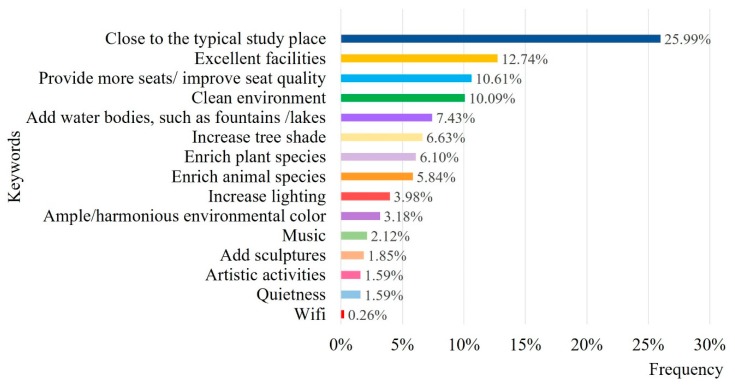
Keyword frequency of ideal attention restoration space on campus.

As shown in Figure 10, compared with other keywords, being close to the typical study place (25.99%) ranked first, followed by excellent facilities (12.74%), provision of more seats (10.61%) and clean environment (10.09%). Students reported that they would like lakes or large open lawns near the library or primary study buildings. The campus environment should be clean, with no leaves on the benches and a clean water body. Moreover, they expressed a strong desire for more seats and improved seat quality. The results indicate that students consider accessibility and comfort to be crucial perceptual factors for the ideal attention restoration space.

Accessibility, visibility, comfort, recognition and sense of belonging were the primary perceptual factors of campus attention restoration spaces, as determined through the analysis of correlations and keyword frequency based on the interaction between the environmental behavior preferences of users and spatial perceptual factors. They were not ranked because the preferences of students on the restorative space were affected by the overall consideration of landscape-type preference and perceptual factors.

### 3.3. CATREG Model of the Design Factors

After the selection of 5 primary perceptual factors, the CATREG model was processed in the following formula:

Y = β_1_X_a_ + β_2_X_v_ + β_3_X_r_ + β_4_X_c_ + β_5_X_s_ + ε (2)

where Y is the recovery degree of selected landscape types; X_a_ is accessibility, X_v_ is visibility, X_r_ is recognition, X_c_ is comfort and X_s_ is sense of belonging, β_1_ to β_5_ are the regression coefficients and ε is an error vector.

Table 4 shows the coefficient of determination R^2^ and the adjusted R^2^ are high, which indicates that there is a good of fit of the regression equation. Also, the correlation between independent variables and dependent variables is significant and the effect of the model is good. For example, as for vegetation space, R^2^ = 0.776, adjusted R^2^ = 0.749, which implies that almost 75% of the variance is explained by the optimal attributes. As for the courtyard space, R^2^ = 0.970, adjusted R^2^ = 0.955, which implies almost 96% explanatory power of its model (Table 4).

In the ANOVA analysis, the F value is large (the smallest F of the four types is 19.20) and the *p*-value of 0.000, together indicate statistical significance of the model. All tolerance is larger than 0.2 which indicates that there is no collinearity. The non-significant factors were removed with the sig-value greater than 0.05 to modelling the optimal-scale regression equation. The classification and regression models of the standardized coefficients of four landscape types and primary perceptual factors are respectively obtained as shown below:

Y_ws_ = 0.998X_r_ (3)

Y_vs_ = 0.194X_a_ + 0.302X_v_ + 0.245X_r_ + 0.193X_c_ + 0.37X_s_ (4)

Y_cs_ = 0.565X_r_ + 0.498X_c_ (5)

Y_ss_ = 0.37X_a_ + 0.361X_v_ + 0.361X_c_ + 0.529X_s_ (6)

where Y_ws_ is waterfront space, Y_vs_ is vegetation space, Y_cs_ is courtyard space, Y_ss_ is square space, X_a_ is accessibility, X_v_ is visibility, X_r_ is recognition, X_c_ is comfort and X_s_ is sense of belonging.

The Standard regression coefficient reflects each explanatory variable. It can be seen that the recognition of 0.998 has the most significant effect on waterfront space from model (3). The five main perceptual factors all have significant influence on vegetation space from model (4). Recognition and comfort show significant influence on courtyard space from model (5). Accessibility, visibility, recognition and sense of belonging are influential factors of square space from model (6).

The relative importance proposed by Pratt (1987) from CATREG explains the percentage of contribution of the independent variables [42]. As shown in Table 4, recognition is the principal factor on waterfront space (99.8%). The perceptual factors rank of vegetation space was sense of belonging (29.7%), visibility (24.7%), recognition (18.1%), accessibility (14.6%) and comfort (13%). The rank of courtyard space was recognition (38.1%) and comfort (26.7%). And the rank of square space was sense of belonging (40.9%), comfort (24.5%), accessibility (20.5%) and visibility (20%).

## 4. Discussion

### 4.1. Optimal Selection of Landscape Types based on Environmental Preference

It would be effective to create an attention restoration space with more preferred spaces, given campus conditions. Water exhibited high desirability among the landscape elements for restoration [43]. Water should be a prioritized landscape type when designing a space, although it could be limited by economic conditions, the geographical location of the campus, water resources and climate regions. In particular, large open water areas were more preferred on the campus map. The results from the campus mapping suggested that vegetation spaces with large open lawns surrounded with tall trees are highly preferred spaces. Academic courtyards can employ a healing garden concept in a compact campus [44]. However, courtyards with open windows or square spaces without any shelter are the least preferred spaces. Students reported that they would enjoy squares if shade and seats are provided. Natural elements could be employed to improve such spaces. For example, the arrangement of deciduous trees in courtyards may provide privacy as well as reduce the direct contact of sight from indoor and outdoor.

Four types of spaces on campuses were reported to have restorative potential and the results indicated that environments with natural elements as more restorative than those with artificial elements [45]. For campuses with enclosed spaces, the courtyard spaces may increase overall satisfaction. For campuses with a higher number of individual buildings, improving vegetation spaces in front of the buildings may yield a positive effect. Overall, the priority should be to enhance the spaces along the necessary activity paths of students and integrate the primary perception factors with the design.

### 4.2. Spatial Quality Improvement Based on Primary Perceptual Factors

The primary perceptual factors were treated as exploratory factors to contribute to ART in the research on restorative campus environments. Recognition was in accordance with legibility and sense of belonging was comparable to compatibility in ART [20]. The findings of accessibility were consistent with the previous studies that spaces near nature have improved perceived restorative effects [46,47]. These findings indicate that students consider visibility and comfort to be crucial factors that contribute to restorativeness. Moreover, the study contributes to the ongoing debate on healthy planning by indicating that the quality of the environment and seats must be a priority. The primary perceptual factors are discussed in spatial dimensions in the following section to assist future research and design.

Table 5 shows an improved pattern of the five primary perceptual factors. The layout of the attention restoration space should be designed within an acceptable walking distance to encourage access and be located on a path where frequent activity happens [48,49]. Visibility should be enhanced through introducing good lines of sight from different perspectives, as well as a high green looking ratio [50]. Sufficient landscape space should be reserved around the library and teaching buildings to ensure the continuity of green spaces, water bodies and visual openness for future campus planning. The facilities and the neatness of the environment should be improved to enhance the comfort factor [51]; providing movable seats for students and avoiding cold materials or windward locations would be worthwhile. Lighting at night should be enhanced in the necessary activity spaces because night is the peak preferred usage time. A clear layout pattern with a coordinated and unified design can enhance recognition [22], which may include making clear guidance to the accessible space using similar design approaches and improving spatial coherence through road planning. The demand of users for the sense of belonging could be achieved through designing a place they feel safe [52,53]. It is interesting to note that the need for places that allow a person to see but without being seen was consistent with the prospect-refuge theory developed by Appleton [54]. For instance, setting up shelters to provide privacy could be beneficial.

### 4.3. Comprehensive Evaluation of Key Design Factors

In this study, the primary design factors of attention restoration spaces are landscape types and perceptual factors, which interact with each other. The evaluation of their advantages and disadvantages based on the actual situation of different campuses is crucial (Table 6). Therefore, the design factors must be analyzed using the CATREG model.

Waterfront spaces are seldom affected by accessibility, visibility, comfort and sense of belonging. The higher the recognition is, the higher the restorative score of the waterfront space is. Accessibility is an inferior element for waterfront spaces (Table 6) because most students reported that the distance between waterfront spaces and learning spaces was far. The other three perceptual factors could be considered to be second options in the specific design.

Vegetation spaces on campuses are usually the most common landscape types; however, the ones students perceived to be restorative must follow the primary perceptual factors. Sense of belonging suggests that the open green spaces can provide some shelters, which is in accordance with the campus mapping result of a large lawn with trees. Visibility is significant because of the preference for relaxation through taking in views through windows and the restorative effect is primarily a result of the green vision rate [55]. Therefore, improving visibility near buildings and along the frequently visited routes is essential. The importance of recognition, accessibility and comfort are similar, as determined through the CATREG model and should be considered according to the campus situation.

The courtyard spaces have an enclosure layout, thus providing a sense of belonging, which could be a dominant factor. People could enjoy the view of the courtyard from the window and from the surrounding buildings, which indicates that effects on visibility could be weak. Moreover, reaching the courtyard can be easy for students. Therefore, recognition and comfort should be improved when considering the users around the courtyard.

Square spaces have a strong sense of form with a large open space of pavement and thus the recognition influence is weak. The other four primary perceptual factors can be improved. Among these factors, the sense of belonging has the strongest influence because of the lack of shelter.

The result of “importance” in the CATREG model provides an optimization scheme for the design of attention restoration spaces based on the student requirements for landscape types. The model suggested that all factors are crucial and only the remaining variables are most salient in decision making concerning spatial designs.

### 4.4. Limitations and Future Research

This study examined the attention restoration spaces based on the evidence of the environmental preference of students for using campus landscapes as a space to restore attention. However, this study has limitations, which can be further examined in future research. The sample campuses were in northeastern China and thus are affected by cold seasons. Therefore, the concerns and needs of university students may differ from those in other climate regions. Future research should examine seasonal factors. Moreover, similar studies in diverse regions with varied cultural backgrounds, education levels and climate conditions may provide further insights into specific contexts. Further studies can explore specific design elements and seasonal effects, focusing on campus landscapes. Another limitation is the sample size of each campus. Although all campuses were considered as a part of the complete sample to explore the difference between landscape types, the large workload limited the number of collected questionnaires. Future research may include more students using the aforementioned method at certain campuses.

## 5. Conclusions

The study examined attention restoration spaces as essential places for improving the wellbeing of students at university campuses and particularly highlighted the environmental preference of users to improve the quality and potential of the restorative spaces. ART was applied on a modified scale through perceptual factors to investigate perceived restorative spaces. Questionnaires integrated with place-mapping as a visual approach were employed to reveal spatial chrematistics of campus landscapes. The CATREG model was used as a quantitative analysis approach to identify the relationship between landscape types and perceptual factors. The results supported the argument that perceived restorative factors of a campus outdoor environment have a positive correlation with naturalness [56]. The landscape types and perceptual factors affect the restorative usage patterns of the campus outdoor spaces. For the optimal selection of landscape types, waterfront spaces should be given the highest priority, followed by vegetation spaces, courtyard spaces and squares designed with the primary perceptual factors. Visibility, accessibility, comfort, recognition and sense of belonging were the primary perceptual factors and these could reflect the spatial requirements of relaxation for university students at campuses. Therefore, the integration of the primary perceptual factors with the optimal space types is suggested as well as making use of the favorable factors, improving weak factors and selecting factors that can be easily realized. Overall, the design implications of attention restoration spaces based on the environmental preferences of users could help landscape designers and school planners create healthy spaces for students. A campus environment with suitable restorativeness can be a crucial part of the public open spaces in cities and can supplement urban green spaces. In particular, campuses located in the city center can serve citizens living nearby. This study can be extended to the open spaces in cities and provide a reference for the restorative design of public green spaces.

## Figures and Tables

**Table 1:** Information of the university samples.

Sample	University	Area (hm^2^)	Green Coverage Rate	Campus Built Time	Landscape Types
1	Northeast Normal University (main campus)	47	21%	1950	SS, CS, VS, WS
2	Northeast Normal University (Jingyue campus)	50	38%	2002	SS, CS, VS, WS
3	Jilin University (new campus)	170	33%	2015	SS, CS, VS, WS
4	Jilin Jianzhu University	41.7	28%	2008	SS, CS, VS
5	Harbin Engineering University	125.61	14%	1994	SS, CS, VS, WS
6	Northeast Forestry University	136	27%	1985	SS, CS, VS, WS

Note: SS: Square space, CS: Courtyard space, VS: Vegetation space, WS: Waterfront space.

**Table 2:** Perceptual factors of attention restoration space on the university campus.

No.	Perceptual Factors	Definitions
1	Accessibility	Easy to reach, no obstacles in space and time.
2	Visibility	The scope of landscape elements that can be seen, including spatial scale and openness.
3	Recognition	Easy to find the place and the composition of the layout is easy to understand.
4	Familiarity	The environmental characters are familiar and accessible.
5	Comfort	A comfortable environment with good landscape maintenance, such as seating, paving, lighting, etc.
6	Pleasure	Feel happier with the landscape design or the activities happened here.
7	Sense of belonging	Feel safe and feel like belong here.
8	Quietness	A sense of tranquility; the environment is quite or there are natural or pleasant sounds.
9	Exploration	Attractive and raise curious.
10	Creativity	Stimulate creative thinking.

**Table 3:** Correlation between perceptual factors and user preference of attention restoration space (n = 360).

Perceptual Factors	Correlation Coefficient	Sig.	Correlation Coefficient	Sig.
	Frequency Using Such Space in Good Weather		Average Time Spent in Such Space	
Accessibility	0.125 *	0.018	0.01	0.85
Visibility	0.142 **	0.007	−0.01	0.853
Recognition	0.174 **	0.001	−0.042	0.428
Familiarity	0.103	0.05	−0.065	0.221
Comfort	0.103	0.051	0.112 *	0.034
Pleasure	0.093	0.078	0.091	0.086
Sense of belonging	0.146 **	0.006	0.004	0.943
Quietness	0.096	0.069	0	0.997
Exploration	0.092	0.081	0.01	0.854
Creativity	0.099	0.06	0.064	0.224

Note: ** Correlation is significant at the 0.01 level (2-tailed); * Correlation is significant at the 0.05 level (2-tailed).

**Table 4:** Categorical regression (CATREG) output of four landscape types and five primary perceptual factors (n = 360).

Landscape types	R^2^	Adjusted R^2^	F	Variables	Standardized Coefficients		Sig.	Imp.
					Beta	Std. Error		
Waterfront space	1.000	1.000	9,159,344.82	accessibility	0.004	0.476	1.000	0.002
				visibility	−0.004	0.432	1.000	0.000
				recognition	0.998	0.326	0.000 *	0.998
				comfort	0.000	0.163	0.999	0.000
				sense of belonging	0.001	0.335	1.000	0.000
Vegetation space	0.702	0.681	33.70	accessibility	0.194	0.068	0.005 *	0.146
				visibility	0.302	0.076	0.000 *	0.247
				recognition	0.245	0.071	0.000 *	0.181
				comfort	0.193	0.071	0.000 *	0.130
				sense of belonging	0.370	0.120	0.000 *	0.297
Courtyard space	0.953	0.930	42.53	accessibility	0.420	0.249	0.064	0.292
				visibility	0.256	0.216	0.269	0.086
				recognition	0.565	0.253	0.038 *	0.381
				comfort	0.498	0.260	0.045 *	0.267
				sense of belonging	−0.055	0.175	0.757	−0.026
Square space	0.844	0.800	19.20	accessibility	0.370	0.105	0.000 *	0.205
				visibility	0.361	0.100	0.001 *	0.200
				recognition	−0.177	0.100	0.054	−0.058
				comfort	0.361	0.099	0.001 *	0.245
				sense of belonging	0.529	0.098	0.000 *	0.409

* Significance at level of significance = 0.05.

**Table 5:** Primary perceptual factors design diagram of the attention restoration space.

Perceptual Factors	Accessibility	Visibility	Comfort	Recognition	Sense of Belonging
Negative space example	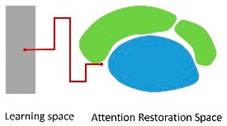	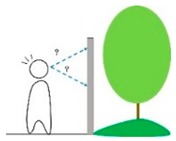	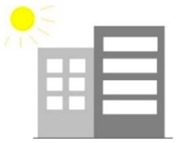	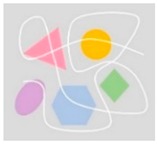	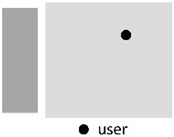
Positive space example	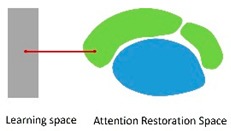	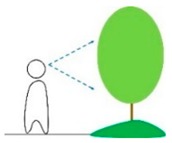	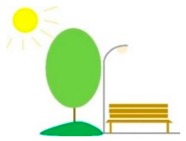	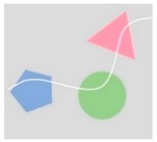	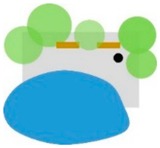

Note: The illustrations only serve as the understanding of perceptual factors.

**Table 6:** Evaluation of the advantages and disadvantages based on CATREG model.

Landscape Types	Primary Perceptual Factors	
	Advantages	Disadvantages
WS	visibility, comfort, sense of belonging	accessibility, recognition
VS	-	accessibility, visibility, recognition, comfort, sense of belonging
CS	visibility, accessibility, sense of belonging	comfort, recognition
SS	recognition	visibility, accessibility, comfort, sense of belonging

Note: WS: Waterfront space, VS: Vegetation space, CS: Courtyard space, SS: Square space.
